# Medical Association of Central New York

**Published:** 1900-12

**Authors:** C. A. Vander Beek


### Society Proceedings.

### MEDICAL ASSOCIATION OF CENTRAL NEW YORK.

REPORTED BY C. A. VANDER BEEK, M. D., Secretary.

THE thirty-third annual meeting of the Medical Association of Central New York was held at the Genesee Valley Club, Rochester, N.Y., on October 16, 1900.

The meeting was called to order at 11.00 a.m., by Dr. JOHN GERIN, president.

Dr. VAN DER BEEK, Secretary.—Inasmuch as the minutes of the preceding meeting have been stenographically reported and printed in the transactions, I would move that the reading of the minutes be dispensed with. Seconded. Carried.

The president appointed the following committees:

*Credentials.*—A. L. Beahan, Ontario; W. J. Herriman, Monroe; J. C. Fisher, Chemung.

*Business.*—D. M. Totman, Onondaga ; J. P. Harley, Cayuga; F. S. Crego, Erie.

*Reception.*—C. A. Van der Beek, E. B. Angell, W. S. Ely, T. Oliver Tait, Wm. M. Brown, Monroe.

*Ethics.*—Marion Craig Potter, Monroe; Charles H. Richmond, Livingston; M. L. Bennett, Schuyler.

*Publication.*—Wm. C. Krauss, Erie; C. A. Van der Beek, S. L. Elsner, Monroe.

*Legislation.*—John W. Whitbeck, E. V. Stoddard, Monroe; Nathan Jacobson, Onondaga; John Gerin, Cayuga; C. M. Lee, Oswego.

The PRESIDENT.—The committee appointed last year to take into consideration the advisability of changing the name of this Association, I believe, is prepared to report.

Dr. E. B. ANGELL, Monroe.—The committee was asked to report upon three points:

First, with reference to the advisability of changing the name of the Association, and if it was so desirable, substituting for the name "Medical Association of Central New York" the title, "Western New York," or "Central and Western New York."

Second, with reference to the date of the meeting. It seems that the date of the meeting, the third Tuesday in October, is the same date as the beginning of the State Medical Association. Some of our members wish to attend that meeting, and dislike being absent from this, therefore they asked that the date might be changed.

Third, the treasurer suggests that some measure has got to be adopted in order to replenish the treasury within the next year or so, at least, and the Committee was desired to make some recommendation with reference to that point.

Your committee has had a meeting and makes the following recommendations:

1. That the name of the Association be changed to "The Medical Association of Central and Western New York."

2. That the date of the meeting be changed to the fourth Tuesday in October.

3. That the Treasurer be directed to send bills for annual dues to all permanent members.

(Signed) NATHAN JACOBSON.
EDW. B. ANGELL.
WM. C. KRAUSS.

The fourth Tuesday in October does not conflict with the dates of any County Medical Associations, or any of the State Associations or National Associations, so far as your committee can find out. Therefore, your committee recommends that the date of the meeting be changed to that of the fourth Tuesday in October instead of the third Tuesday in October.

With reference to the dues, the committee recommends that the treasurer be directed to send bills for annual dues to all permanent members, whether they are present at each meeting or not. It has been the custom, I understand, in the past, to ask only those who attend the meeting to pay the annual dues of $1.00. Inasmuch as the Association has undertaken the burden of publishing the transactions each year and sending a copy of such transactions to each member who pays annual dues, it is considered only proper that each permanent member pay his dues and receive his copy of the transactions. Therefore this recommendation is made, that the treasurer be directed to send bills for the annual dues to all permanent members. That does not include the delegate members, only the permanent members.

With reference to the penalty that shall follow nonpayment of dues, that will be well to leave for the future to determine how successful the treasurer shall be by simply sending out his due bill.

Therefore, Mr. Chairman, these are the recommendations made by the committee. The first two recommendations are changes in the constitution, and notice will have to be given of such changes, and will lie on the table for one year. The third recommendation is more in the nature of a by-law, and I understand the society is competent to adopt that at any time.

Therefore, I give notice that I will move at the next meeting the adoption of these two suggestions with reference to changes in the constitution at the next meeting.

The third recommendation, with reference to sending bills out to all permanent members for annual dues, I believe the society can take action upon.

Dr. F. B. SEARS, Onondaga.—I move the report be adopted. Seconded. Carried.

The President, Dr. JOHN GERIN, of Auburn, then read his address on Electricity.

The following papers were read and discussed:

Autoinfection as a factor in diseases of infants and children, J. Roberts Johnson, Syracuse.

Report of a case of sarcoma of the tonsil and its removal by external pharyngotomy, Nathan Jacobson, Syracuse.

How eye-strain causes headache, Lucien Howe, Buffalo.

Empyema, and its treatment in children, M. L. Bennett, Watkins.

Some mistaken impressions regarding troubles of the genito-urinary organs, J. Henry Dowd, Buffalo.

The longevity of the gonococcus, E. Wood Ruggles, Rochester.

The association adjourned for luncheon at the invitation of the Monroe County Medical Society.

AFTERNOON SESSION.

Meeting called to order by the president at 3.25 p.m.

Treasurer's report read and accepted.

The PRESIDENT.—Are there any other committees to report?

Dr. E. B. ANGELL, Monroe.—I wish to make a supplementary report from the special committee appointed last year, and that is to this effect, that in discussing the matter with some of our Buffalo members, it has been deemed quite advisable to hold a meeting of two days' duration next autumn. It seems that the meeting is to occur in Buffalo, in accordance with the regular program, and that Buffalo is to hold a large exposition next year. The Buffalo men desire to show us something of the city and something of the exposition, as well as to have us listen to papers. Therefore, they have asked, or it has been suggested that they hold a two days' meeting, and for that reason, the committee recommends a suspension of the rules and by-laws of the association for the coming year, so as to enable the Buffalo committee to arrange for this meeting at such date in the autumn as they may find expedient.

The date according to the one suggested by the committee would be the fourth Tuesday in October, if that is adopted, and that is rather late in the autumn for the exposition, which closes the 1st of November. For that reason, it would be wiser to have the meeting occur earlier in the autumn, perhaps the last of September or first of October, so that this suggestion is made, that the Association suspend the by-laws for this next year. I make that as a motion, so that the committee of arrangements in Buffalo can arrange for the meeting at such date as they find most expedient, to be announced by that committee.

Seconded by Dr. Wm. Brown, Monroe. Carried.

The President.—The committee, as reported by Dr. Angell this morning, made certain recommendations, which contemplate a change in the name of the Association. Doctor, would you please state those recommendations again?

Dr. E. B. Angell, Monroe.—The first recommendation which must be taken up, of course, now, is that the name of the Association be changed to "The Medical Association of Central and Western New York, to take the place of "The Medical Association of Central New York."

Dr. A. L. Benedict, Erie.—I would like to amend by substituting the word "Society" for "Association." Seconded.

Dr. E. B. Angell, Monroe.—I don't see that we are going to gain anything by the substitution of "society" for "association." This has been known as the association for the last thirty years, and unless there is good reason for making that change, I should be opposed to it personally.

Though I made that report and moved its adoption, I am opposed to making any change in the name of the association. I don't believe we are going to gain anything by adopting the name of "The Medical Association of Central and Western New York." It is awkward, inconvenient and the association has done very well in its present form. The only change to make would be to call it "The Association of Western New York," which would no longer identify it with the old association, and this organisation, founded by Dr. E. M. Moore, of Rochester, and Dr. Didama, of Syracuse, goes by too good a name to have it altered. I am opposed personally to any change.

Dr. F. S. Crego, Erie.—I think if there is to be a change, we should make it "The Society of Central and Western New York," so that it may not conflict with "The Central-Western New York Medical Association," which is a branch of the State Association. The most of us are affiliated with the State Society, and I think, if you change at all, we ought to make a complete change, so as not to get mixed up with the State Association. However, I think Dr. Angell's suggestion is very good, to leave it as it is.

Dr. Wm. C. Krauss, Erie.—At the Auburn meeting, two years ago, I think, I suggested a change to "Central and Western New York," because all the county societies in western New York are now membeis of this association; and with the exception of a few counties, east of Monroe, we haven't any membership; it seemed to me at that time but just and right to include those western counties in this association, and then to change the name to "Central and Western New York." I think the change of the word "association" to "society" is not advisable, and I will stand by the report of the committee.

Dr. A. L. Benedict, Erie.—I would like to say this, that personally, I agree with Dr. Angell, that it is better not to change at all; but if we do change, we ought to use the word "society," because our delegates come from the county societies, and as it has been said, we are more affiliated with the society than the association, and, unfortunately, the two words are not entirely synonymous. If we make this change at all, we ought to do it in keeping with the nomenclature, especially as long as county associations are being formed as well as county societies.

Dr. Wm. Brown, Monroe.—I feel very much as has been expressed by Dr. Angell and Dr. Crego, that the name of the society is very well let alone, and I would make a motion, that the matter be laid on the table indefinitely. Seconded. Carried.

Dr. E. B. ANGELL, Monroe.—The second change recommended by the committee was with reference to the change of date. It was recommended that we meet on the fourth Tuesday in October rather than the third Tuesday in October. The third Tuesday in October is late enough, and I believe that an earlier date than that would be inadvisable. Therefore, personally, I am opposed to the change; while, for the sake of bringing it before you, I move that the recommendation of the committee be adopted.

Dr. F. B. SEARS, Onondaga.—Inasmuch as next year is left to the discretion of the committee, I move that that be laid over until then, when we will know more about it. Seconded. Carried.

Dr. E. B. ANGELL, Monroe.—The further recommendation was with reference to the matter of collecting the dues. It was recommended that the treasurer send a bill of dues to each permanent member of the association hereafter, in place of only requiring those to pay who are present at the meetings. The adoption of that, I move. Seconded.

Dr. A. A. YOUNG, Wayne.—I would like to ask if the payment of those dues are to be made obligatory or not; and whether, if not paid, the names of the members would be dropped from the roll?

Dr. E. B. ANGELL, Monroe.—That has not been included in the motion. That will be for the further consideration of the society at a later date. We will simply see how this will work.

Dr. C. A. VAN DER BEEK, Monroe.—I would like to say that the expenses of the secretary have increased from about $28, five years ago, to about $59.00 now. If the expenses of the treasurer increase in like propertion, I think we will have to assess each one $2.00 instead of $1.00.

Dr. E. B. ANGELL, Monroe.—I would further explain that when the association started to publish its transactions, it had $280.00 or $290.00 in the treasury. That has been gradually used up, there being debts each year, until the capital of the association last year, at the meeting in Syracuse, was $74.00. It so happens that this year, we are a little ahead, but that doesn't always follow. We have got to publish shortly a copy of the constitution and by-laws and a list of the members, which will require a large amount of money, and it is for this reason, I think, that we ought to be forewarned in regard to the possibility of having an empty treasury. It is true that if any member who is not present at the Association will forward a dollar, he will get a copy of the transactions.

Dr. S. L. Elsner, Monroe —In giving the report, there was a balance of $124.00, but two bills came in today, which foot up between $58.00 or $59.00. Those were not put in that report. That would leave really a balance of about $70.00 in the treasury; and it is a fact that the expenses are growing heavier every year; and the registration, though large today, has not been this size, nor has the same amount of mone y been taken in at any former meeting in some time.

Motion of Dr. Angell carried.

Dr. E. B. Angell, Monroe. —I am sorry that the Syracuse member of the committee was not present, but the Buffalo member, Dr. Wm. C. Krauss, and myself, in discussing the matter, believed it would be unwise and inexpedient for the society to meet at all in Auburn, except by special invitation. I am very sorry to come to that conclusion, not because we were not well entertained in Auburn, but because it is a very hard place to reach. Therefore, I give notice that at the next meeting, I will move an amendment to Article II. of the constitution, so that hereafter we shall meet annually, in turn, at Buffalo, Syracuse and Rochester.

The President.—The secretary suggests that authority be given for publication of a revised constitution and by-laws, also a membership list. The Secretary might perhaps say something on that subject.

Dr. C. A. Van der Beek, Monroe.—I would say that the Secretary has just three copies of the constitution and by-laws and applications are being made frequently for copies which the secretary cannot supply. Now, there are going to be some changes made, I believe, in the by-laws, or in the constitution. I would make as a motion that the publication committee be authorised to have a new copy of the constitution and by-laws printed, which will include a revised list of the members.

Dr. Wm. C. Krauss, Erie.—Inasmuch as changes have been proposed in the constitution, I think it would be well to defer this for another year. Then we will have the constitution revised to date. Notice has been given today of an amendment.

Dr. W. J. Herriman, Monroe.—I beg pardon for not rising earlier, before you started, but notice has been given us today that at the next meeting of the association, an amendment to the constitution will be offered, changing the name of the association. Whether the amendment will go through or not at the next meeting, it seems to me that that is sufficient reason for deferring the publication of the new constitution and by-laws until another year and until action shall be taken upon that proposed amendment.

Dr. C. A. Van der Beek.—I think Dr. Herriman and Dr. Krauss are right, and I withdraw the motion.

The election of officers for the ensuing year resulted as follows: President, Lucien Howe, Erie; vice-president, A. L. Beahan, Ontario; second vice-president, Ezra B. Potter, Monroe. The secretary and treasurer hold over until next year.

Dr. C. A. Van der Beek.—I would move the election to permanent membership of Drs. F. A. Farshee, Cortland; E. P. Ballantine, J. S. Berkman, C. A. Greenleaf, J. M. Ingersoll, M. E. Leary, E. G. Nugent, J. D. Ozmun, A. C. Remington, J. H. Sullivan, Cornelia White-Thomas, A. W. Thomas, Geo. H. White, Monroe; H. C. Baum, A. W. Hedden, G. G. Lewis, C. E. McClary, R. C. McLennan, G. M. Price, A. S. Ruland, F. W. Slocum, Onondaga; D. S. Allen, Wm. A. Howe, Ontario; Harriet M. Doane, Oswego; R. E. Doran, Seneca, all of whom have complied with the requirements. Seconded. Carried.

The reading of papers was then resumed :

Presentation of a case, S. L. Elsner, Rochester.

Syphilis of the nervous system, Floyd S. Crego, Buffalo.

New method of quantitating proteids in gastric digestion, A. L. Benedict, Buffalo.

Bromides in epilepsy, A. Pierce Clark, Sonyea.

A report at the Buffalo laboratory in the investigation of cancer, Henry R. Gaylord, Buffalo.

Cirsoid aneurism of the ear, Wm. S. Cheesman, Auburn.

Abdominal drainage, A. L. Beahan, Canandaigua.

Some points in the toilet of the peritoneum, Charles E. Condgon, Buffalo.

Asthma in its relation to localised disease of the nose, John O. Roe, Rochester.

Dermatitis exfoliativa, Wm. A. Howe, Phelps.

Report of a case of fibro-myxoma of the abdominal wall, with exhibition of the tumor, Thomas Jameson, Rochester.

Fitting trusses, M. A. Veeder, Lyons.

The following papers were read by titles :

Surgical treatment of injured nerves, E. B. Angell, Rochester.

Alcoholism, F. E. Fronczak, Buffalo.

Importance of sugar in disease, Charles H. Richmond, Livonia.

Formalin, A. A. Young, Newark.

Vivious tendencies in therapeutics, Brace W. Loomis, Syracuse.

Tubercular disease of the brain, Wm. C. Krauss, Buffalo.

The importance of the position of medical examiner for life insurance, F. W. Maloney, Rochester.

Rheumatism, L. A. Weigel, Rochester.

The relation of rectal disease to chronic constipation, D. H. Murray, Syracuse.

At half past seven o'clock in the evening the members of the association were the guests of the Monroe County Medical Society at a commers, at the Genesee Valley Club House, and enjoyed the hospitalities thoroughly. The glee club of the University of Rochester aided not a little in making the evening a most pleasant and enjoyable one.

